# Antioxidant Peptides from Goat Milk Fermented by *Lactobacillus casei* L61: Preparation, Optimization, and Stability Evaluation in Simulated Gastrointestinal Fluid

**DOI:** 10.3390/nu10060797

**Published:** 2018-06-20

**Authors:** Guowei Shu, Xiaoyu Shi, Li Chen, Jianbo Kou, Jiangpeng Meng, He Chen

**Affiliations:** 1 School of Food and Biological Engineering, Shaanxi University of Science and Technology, Xi’an 710021, China; shixiaoyu419@163.com (X.S.); lcshgw@gmail.com (J.K.); chenhe419@gmail.com (H.C.); 2 College of Food Engineering and Nutritional Science, Shaanxi Normal University, Xi’an 710119, China; 3 Department of Research and Development, Xi’an Baiyue Gaot Milk Corp., Ltd., Xi’an 710089, China; byjpmeng@gmail.com

**Keywords:** goat milk, antioxidant peptides, *Lactobacillus casei* L61, response surface methodology, simulated gastrointestinal digestion, scavenging rate of hydroxyl radical, DPPH

## Abstract

Antioxidant peptides are currently the focus of many studies, since they eliminate free radicals in the human body without harmful effects. In the present study, *Lactobacillus casei* L61 was used as a starter culture to ferment goat milk because of its high capacity to produce antioxidant peptides. An optimal nutrients formula (casein, casein peptone, glucose, soybean peptone, inulin, calcium lactate, and cysteine) was investigated by Plackett–Burman (P–B) and Box–Behnken (B–B) designs for response surface methodology (RSM). Antioxidant peptides were successively isolated and purified from the fermented goat milk. Furthermore, the stability of the antioxidant peptides was evaluated in a simulated gastrointestinal tract at 37 °C. The results showed that calcium lactate, glucose, and casein peptone significantly affected the antioxidant activity of goat milk. The optimal additive amounts were 0.99% (*w*/*v*) calcium lactate, 0.21% (*w*/*v*) glucose, and 0.29% (*w*/*v*) casein peptone. The hydroxyl free radical scavenging rate increased significantly (*p* < 0.001) from 56.50 ± 0.57% to 88.01 ± 0.69%; the 1,1-diphenyl-2-picrylhydrazyl (DPPH) radical scavenging rate increased up to 63.48 ± 1.22% under the optimal conditions (*n* = 3). Our research provides a fitted mathematical model for antioxidant peptides production. Besides, these antioxidant peptides had great stability during simulated gastrointestinal digestion.

## 1. Introduction

Free radicals are generated inevitably as by-products during aerobic metabolism, generally as reactive oxidant species (ROS) [1]. The most common ROS in vivo are superoxide, hydroxyl radical, peroxyl radical, nitric oxide, and peroxynitrite [2]. As a result of the oxidation of excessive free radicals, a series of free-radical chain reactions are triggered, causing oxidative damage to proteins, enzymes, lipids, and nucleic acids in the body [3]. This will have a harmful impact on organs and tissues [4], promoting heart disease, diabetes, cancer, and cataracts.

At present, antioxidants are effective compounds for scavenging free radicals and have been widely used in many fields. Studies have shown that supplements which are rich in antioxidant activity can reduce oxidative damage in the human body [5]. However, artificial antioxidants, such as butylated hydroxyanisole (BHA), butylated hydroxytoluene (BHT) and *t*-butylhydroquinone (TBHQ) probably have potential health hazards to the human body [6]. Because of their strong antioxidant activity and high safety, natural antioxidants have gradually attracted considerable attention.

As facultative anaerobic microorganisms, lactic acid bacteria (LAB) were generally investigated for their response to oxidative stresses, the ability to remove radicals, and the production of antioxidant enzymes in cells grown anaerobically [7]. Besides, various bioactive peptides have been isolated from protein hydrolysates fermented by LAB, which were proved to be functional in health promotion. The production of peptides by a hydrolytic reaction is the most promising technique, since peptides have substantially higher antioxidant activity than intact proteins [8]. Goat milk is rich in various protein activities, with an unparalleled advantage. The proteins, fats, minerals, vitamins, and other nutrients of goat milk provide many benefits compared to other types of milk [9,10,11]. Moreover, goat milk overall protein particles are smaller, and its fat is composed of short-chain fatty acids. It is closer to human breast milk than other milk types, and has even a higher level of immunoglobulin than breast milk [12]. So far, 28 small peptides with antioxidant activity have been identified from goat cheese by Sommerer et al. [13]. A research by Li et al. [14] described the preparation, purification, and identification of antioxidant peptides from goat milk casein. Five novel oligopeptides were identified. Two milk protein-derived peptides with sequences of VKEAMAPK and HIQKEDVPSER have been identified from cheddar cheese fermented by *Lactobacillus casei* ssp. casei 300 [15].

In our previous work, *L. casei* L61 was selected as the starter of antioxidant peptides among four probiotic *Lactobacillus* strains. The factors (casein, casein peptone, glucose, soybean peptone, inulin, calcium lactate, and cysteine) affecting the antioxidant activity of *L. casei* L61 in fermented goat milk were investigated by single factor experiments (data unpublished). In this study, we screen and optimize a nutrients formula for goat milk fermentation, aiming to promote antioxidant peptides production by Plackett–Burman (P–B) and Box–Behnken (B–B) design for response surface methodology (RSM). Subsequently, the antioxidant peptides are isolated and purified from goat milk fermented by *L. casei* L61 and exposed to simulated gastrointestinal fluid during different times for stability evaluation.

## 2. Materials and Methods

### 2.1. Strain

*L. casei* L61 was provided by the college of Food and Biological Engineering, Shaanxi University of Science & Technology (Xi’an, China). The starter cultures were stored at −20 °C in freeze-dried powder by using skim goat milk as a cryoprotective agent. *L. casei* L61 was activated successively three times in rehydrated Man Rogosa Sharpe broth (MRS, Hopebio, Qingdao, China) with a 5% inoculum at 37 °C for 24 h prior to use as starter culture to ferment goat milk.

### 2.2. Preparation of Fermented Goat Milk and Whey Fractions

A starter culture containing *L. casei* L61 was inoculated into 100 mL 12.5% (*w*/*v*) of reconstituted goat milk pasteurized at 90 °C for 15 min with a 5% inoculum and fermented at 41 °C for 16 h. Fermented goat milk was adjusted to pH 3.4–3.6 and centrifuged at 5000× *g* for 15 min at 4 °C to obtain a supernatant. The supernatant was collected, adjusted to pH 8.3, and centrifuged at 5000× *g* for 15 min at 4 °C to obtain the corresponding whey fraction, which was used for the determination of the antioxidant activity.

### 2.3. Determination of Hydroxyl Free Radical Scavenging Rate

The hydroxyl free radical scavenging rate was measured according to the method of Kong et al. [16] with modifications. A sample solution of 0.5 mL was pipetted into a test tube, and 1 mL ferrous chloride solution (0.25 mmol/L) and ferrozine solution (0.5 mmol/L) was added. The sample was placed for 10 min at room temperature after mixing it thoroughly. Distilled water was used as a control. The absorbance at 562 nm was measured in triplicate using a UV–vis spectrophotometer (Shanghai Spectrum Instruments Co., Ltd., Shanghai, China). The value of the hydroxyl free radical scavenging rate was calculated as follows:

hydroxyl free radical scavenging rate = (A_2_ − A_1_)/A_2_ × 100% (1)

where A_1_ is the absorbance of the experiment group, and A_2_ is the absorbance of the control group.

### 2.4. Determination of 1,1-diphenyl-2-picrylhydrazyl (DPPH) Radical Scavenging Rate

The measurement of the DPPH radical scavenging rate was based on the method of Kazuko et al. [17] with modifications. The mixture of 2 mL of DPPH radical solution (0.1 mM, USA Sigma, St. Louis, MO, USA) and 2 mL of samples was selected as the experiment group. The mixture of 2 mL of DPPH radical solution and 2 mL of 95% ethanol was used as the blank group. Also, 2 mL of 95% ethanol and 2 mL of samples were mixed as the control group. Every mixture was kept at 25 °C for 30 min after mixing. Finally, the absorbance of the mixture was measured by a UV–vis spectrophotometer at 517 nm; all the tests were carried out in triplicate. The scavenging rate was calculated as follow:

DPPH radical scavenging rate = [1 − (Ai − Aj)/Ao] × 100%(2)

where Ai is the absorbance of the experiment group, Aj is the absorbance of the control group, and Ao is the absorbance of the blank group.

### 2.5. P–B Experimental Design

Many nutrients can be used as a carbon source, a nitrogen source, inorganic salts, and prebiotics to promote the growth of *L. casei*. Therefore, several nutrients, namely, casein, casein peptone, glucose, soybean peptone, inulin, calcium lactate, and cysteine were selected to conduct the P–B-designed experiment. The experiment of P–B design contained seven factors and three error terms, spanning 12 runs at two levels (a higher level coded as +1, a lower level coded as −1) to determine significant factors for the antioxidant activity of goat milk fermented by *L. casei* L61. The factors and coded levels of the P–B design are shown in Table 1.

### 2.6. Steepest Ascent Experiment

The steepest ascent experiment contained three significant factors, i.e., calcium lactate, glucose, and casein peptone at different levels and was performed to determine the central points of the RSM for goat milk fermented by *L. casei* L61. The antioxidant activity of each group of fermented goat milk was measured in triplicate.

### 2.7. B–B Design of RSM

RSM was employed for further optimization studies by determining the maximum response value and evaluating the main effects, interaction effects, and quadratic effects. A three-variable and three-level RSM was used according to the previous study (Table 2).

### 2.8. Isolation and Purification of Antioxidant Peptides

Fermented goat milk samples with optimal additive amounts of calcium lactate, glucose, and casein peptone were collected and treated to obtain the whey fractions. The supernatants obtained were filtered by ultrafiltration with a cut-off of 3000 Da to isolate the antioxidant peptides. Sephadex G-25 and G-15 were employed to separate and purify the filtrated liquid, using gel filtration chromatography (GFC). The component with the highest activity was collected, freeze-dried, and used to determine the resistance to simulated gastrointestinal fluid.

### 2.9. Resistance to Simulated Gastric and Intestinal Fluids

Simulated gastrointestinal digestion was conducted according to the method of Cruzhuerta et al. [18] with modifications. An amount of 6 mg of the separated components was added to 20 mL of simulated gastric fluid (1 g pepsin was dissolved in 100 mL of deionized water, and 1.64 mL of 0.1 mol/L HCl was added). The samples were incubated for 0 min, 40 min, 80 min, 120 min, 160 min, and 200 min at 37 °C. The reactions were stopped by heating at 80 °C for 5 min. The hydroxyl free radical and DPPH scavenging rates were measured. An amount of 20 mL of simulated intestinal fluid (0.68 g K_2_HPO_4_ and 1 g trypsin were dissolved in 100 mL of deionized water, and the pH was adjusted to 6.8 using a diluted NaOH solution) was added to the samples after incubation in the simulated gastric fluid for 40 min. The samples were incubated for 40 min, 80 min, 120 min, 160 min, and 200 min at 37 °C. The hydroxyl free radical and DPPH scavenging rates were measured after stopping the reactions by heating. All the scavenging rates were measured in triplicate.

### 2.10. Statistical Analysis of the Data

SAS (Version 12.0, SAS Institute Inc., Cary, NC, USA) was used for statistical analysis of the P–B-designed experimental data to identify the significant variables and corresponding coefficients. The coefficient, sum of squares (SS %), and confidence intervals (CI) were evaluated to analyze the antioxidant activity in each trial.

Design Expert software (Version 8.0.5, Stat-Ease. Inc, Minneapolis, MN, USA) was employed for the analysis of the B–B designed experimental data obtained, and a quadratic regression equation was established to analyze the response surface contour and surface plots.

## 3. Results

### 3.1. P–B Experimental Design and Results

The result of the P–B design are shown in Table 3; Y_1_ (%) and Y_2_ (%) are the hydroxyl free radical scavenging rate and DPPH radical scavenging rate, respectively.

X1, X5, X6, X7, X8, X9, X10 represent casein, casein peptone, glucose, soybean peptone, inulin, calcium lactate, and cysteine, respectively, while X2, X3, and X4 are three error terms.

**Figure 1:**
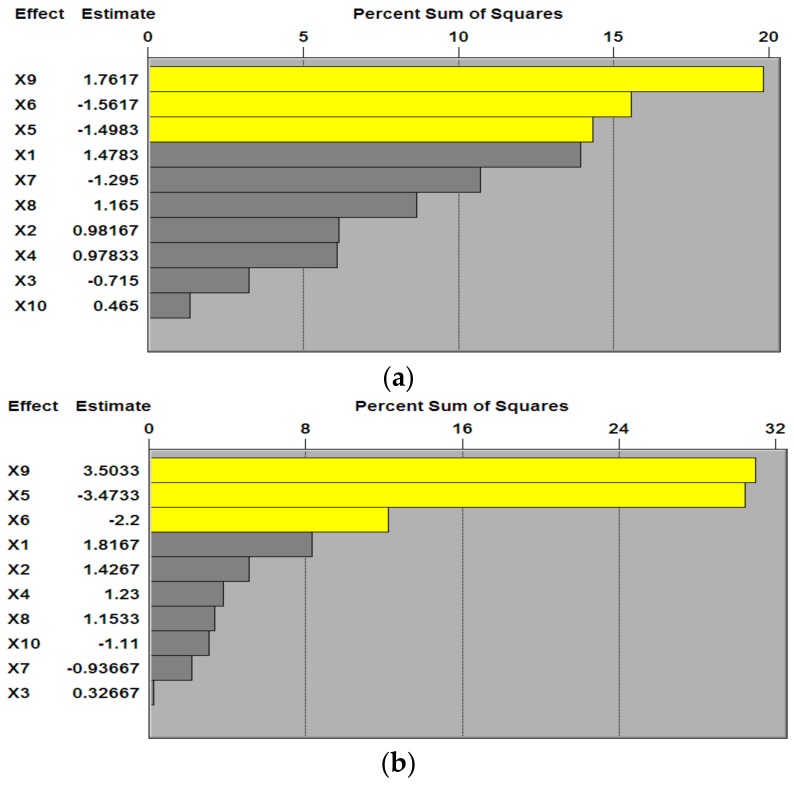
The effect of variable factors on the hydroxyl free radical scavenging rate (**a**) and DPPH radical scavenging rate (**b**).

**Figure 2:**
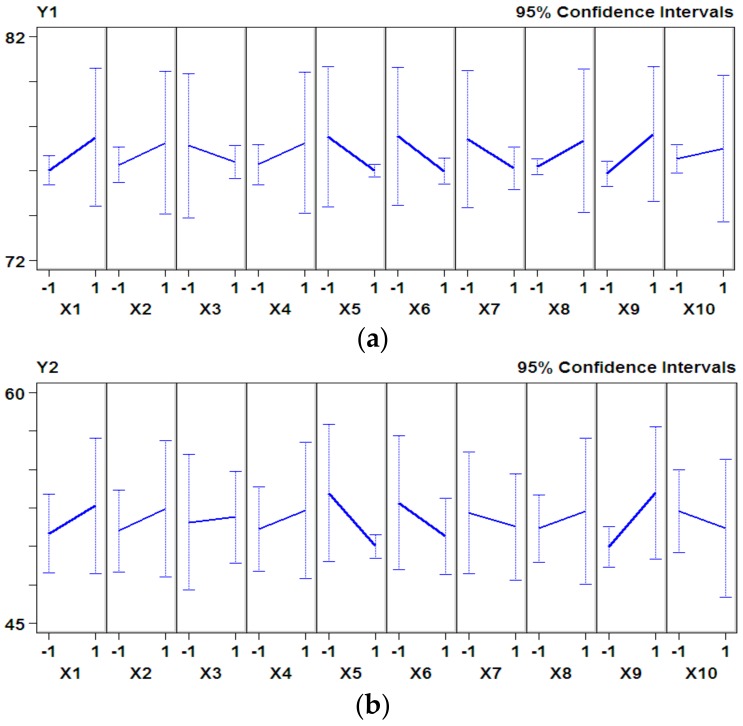
The confidence interval of variable factors for the hydroxyl free radical scavenging rate (**a**) and DPPH radical scavenging rate (**b**).

The effects of nutrients were different during fermentation, therefore the antioxidant activity changed under different conditions. As shown in Figure 1, three variables, i.e., casein peptone (X5), glucose (X6), and calcium lactate (X9), accounted for a large proportion of the percent sum of squares on the Pareto chart for both hydroxyl free radical scavenging rate and DPPH radical scavenging rate. This indicated that the three variables had significant positive effects on the antioxidant activity (Figure 2). According to the results of the P–B design and principal factor analysis, casein peptone, glucose, and calcium lactate were selected as the main factors for further analysis by the steepest ascent experiment; Y1 (%) and Y2 (%) are the hydroxyl free radical scavenging rate and DPPH radical scavenging rate, respectively.

### 3.2. The Experimental Design and Results of the Steepest Ascent Experiment

The steepest ascent experiment was preformed to determine the central points of the RSM. Table 4 lists the design and results of the steepest ascent experiment for nutrients promoting peptide production. Y_1_ (%) and Y_2_ (%) represent the hydroxyl free radical scavenging rate and DPPH radical scavenging rate, respectively.

As can be seen from Table 4, both hydroxyl radical scavenging rate and DPPH radical scavenging rate reached the maximum in the fifth step during the steepest ascent experiments. Therefore, the levels of each factor in step five, which were 1.0% (*w*/*v*) for calcium lactate, 0.2% (*w*/*v*) for glucose, and 0.3% (*w*/*v*) for casein peptone, were used as the center points of the subsequent RSM.

### 3.3. B–B Experimental Design and Results

B–B experimental design and results are shown in Table 5. The hydroxyl free radical scavenging rate is represented by Y_1_ (%), and the DPPH radical scavenging rate is represented by Y_2_ (%).

The data were analyzed to get a quadratic regression model by using Design Export based on the data from the B–B experiment. A multiple regression equation correlating the response function with the independent variables could be obtained as:

Y_1_ = 88.14 − 1.29A + 0.79B − 1.12C + 0.45AB − 2.13AC + 0.21BC − 2.94A^2^ − 1.01B^2^ − 2.16C^2^

Y_2_ = 63.89 + 0.71A − 1.53B − 1.87C − 3.90AB − 3.05AC − 0.87BC − 1.27A^2^ – 4.91B^2^ − 8.51C^2^

where Y_1_ and Y_2_ are the corresponding expected values of the hydroxyl free radical scavenging rate and the DPPH radical scavenging rate, and A, B, and C are the coded values of the independent variables calcium lactate, glucose, and casein peptone, respectively.

Analysis of variance (ANOVA) was used to verify the validity of the model and its parameters according to the significance, as shown in Table 6. Y_1_ (%) and Y_2_ (%) represent the hydroxyl free radical scavenging rate and DPPH radical scavenging rate, respectively.

**Figure 3:**
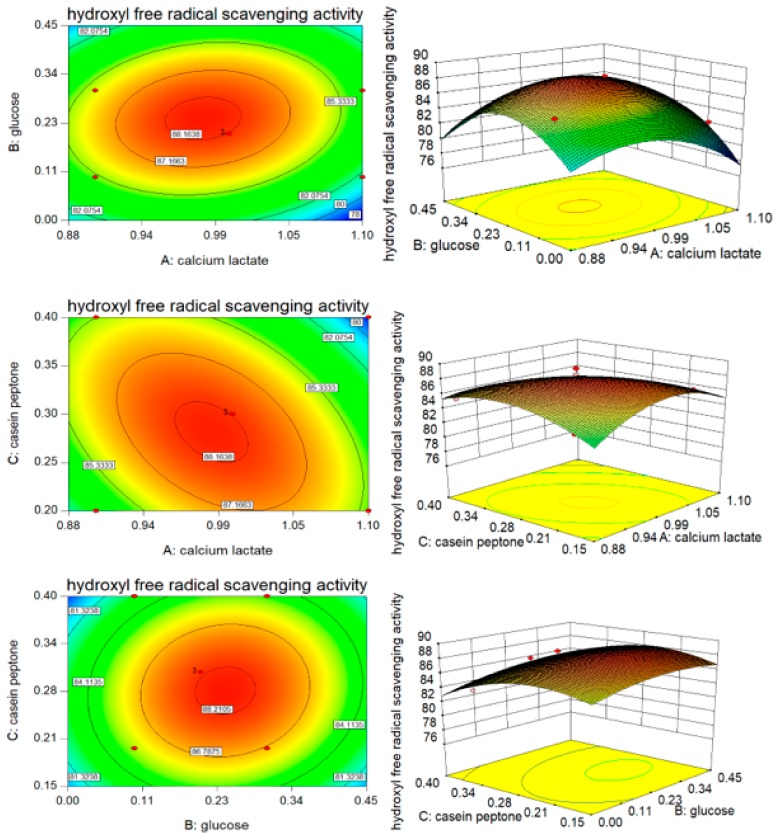
Contour plots and response surface plots of calcium lactate (A), glucose (B), and casein peptone (C) for the hydroxyl free radical scavenging rate (Y_1_).

As shown in Table 6, a significant value for the hydroxyl free radical scavenging rate (*p* = 0.0045 < 0.01) and an insignificant value of the lack of fit (*p* = 0.1835 > 0.05) revealed the effectiveness of the regression analysis, which suggested that the regression model could be used to fit the effect of the three factors on hydroxyl free radical scavenging rate. As the ratio of the explained variation to the total variation, the coefficient of determination (R^2^) can be used to measure the degree of fit. The value of R^2^ was 96.3%. This suggested that 96.3% of the response to the hydroxyl free radical scavenging rate was caused by changing the concentration of A, B, and C and by their interactions. Two-dimensional contours revealed that hydroxyl free radical scavenging rate changed with changes in the temperature, whey powder, and calcium lactate concentration, and their corresponding three-dimensional response surface were generated to better determine the interaction of the three variables with the corresponding variables (AB, BC, AC, Figure 3). The contour plots seemed to be elliptical or nearly circular. This implied that AB, AC, and BC had mutual interactions affecting the hydroxyl free radical scavenging rate, while AB and BC were weak interactors in this respect (*p*_AB_ = 0.3442 > 0.05, *p*_BC_ = 0.6430 > 0.05). In addition, there was not a simple linear correlation between the variables, calcium lactate, casein peptone, and hydroxyl free radical scavenging rate (*p*_A_^2^ = 0.0012 < 0.01, *p*_C_^2^ = 0.0046 < 0.01).

**Figure 4:**
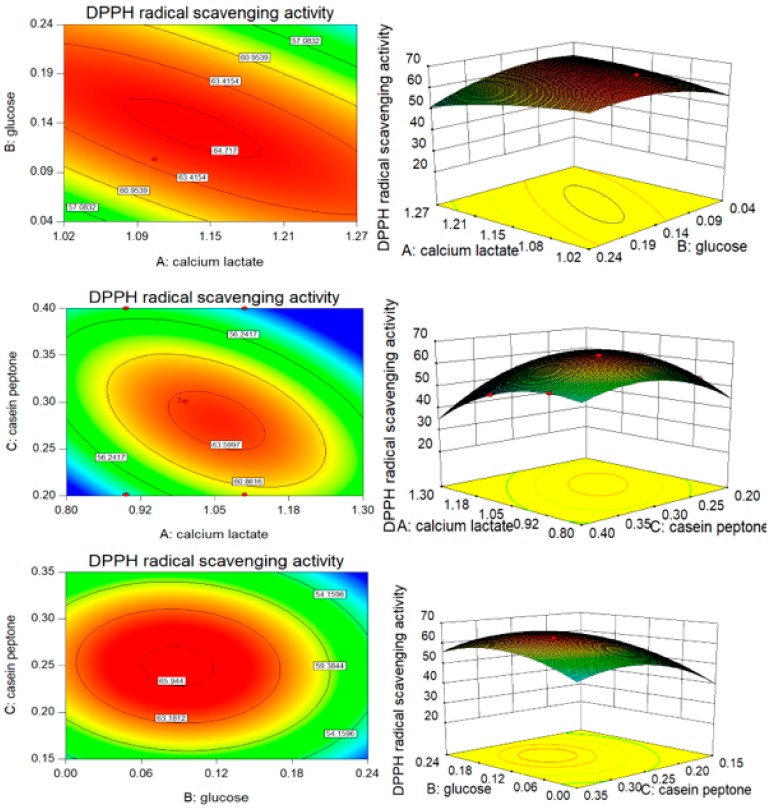
Contour plots and response surface plots of calcium lactate (A), glucose (B), and casein peptone (C) for the DPPH radical scavenging rate (Y_2_).

The ANOVA showed that the model for the DPPH radical scavenging rate had a *p*-value <0.01, which was statistically significant (Table 6). The model equation was corroborated to be a suitable model to describe the value of the DPPH radical scavenging rate, while the lack of fit (*p* = 0.0854 > 0.05) was insignificant. The value of R^2^, which was 97.96%, indicated that only 2.02% of the variability on the DPPH radical scavenging rate could not be explained by the predicted equation of model. The *p*-value of BC was 0.2759, which suggested that A and B had a strong mutual interaction affecting the DPPH radical scavenging rate (Figure 4).

The maximum responses values of the hydroxyl free radical scavenging rate (88.36%) and DPPH radical scavenging rate (63.79%) were obtained at additive amounts of 0.99% (*w*/*v*) calcium lactate, 0.21% (*w*/*v*) glucose, and 0.29% (*w*/*v*) casein peptone as predicted. The design of the verification experiment was dependent on the optimization results (A = 0.99%, B = 0.21%, C = 0.29%). The results demonstrated that the hydroxyl free radical scavenging rate and DPPH radical scavenging rate were 88.01 ± 0.69% and 63.48 ± 1.22% under the optimum conditions (*n* = 3). There was no significant difference from the predicted values, indicating the model was appropriate. The control had a scavenging rate of hydroxyl free radical of 56.50 ± 0.57% and a scavenging rate of DPPH radical of 41.97 ± 0.72% without addition of calcium lactate, glucose, and were peptone (*n* = 3). The hydroxyl free radical scavenging rate and DPPH radical scavenging rate was increased by 31.51% and 21.51%, respectively, after optimization by RSM.

### 3.4. Simulated Gastrointestinal Digestion

**Figure 5:**
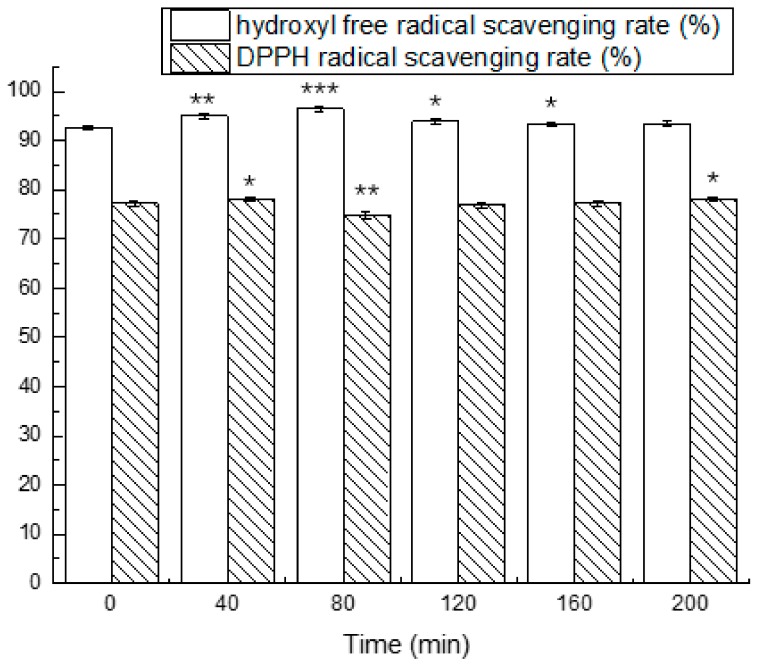
Effects of simulated gastric fluid on the hydroxyl free radical scavenging rate and DPPH radical scavenging rate at different times. The asterisks (*) indicate significant differences (*** *p* < 0.001, extremely significant; ** *p* < 0.01, very significant; * *p* < 0.05, significant.) of antioxidant activity in the simulated gastric fluid during different times compared to 0 min. Mean values (*n* = 3) ± standard error are shown.

**Figure 6:**
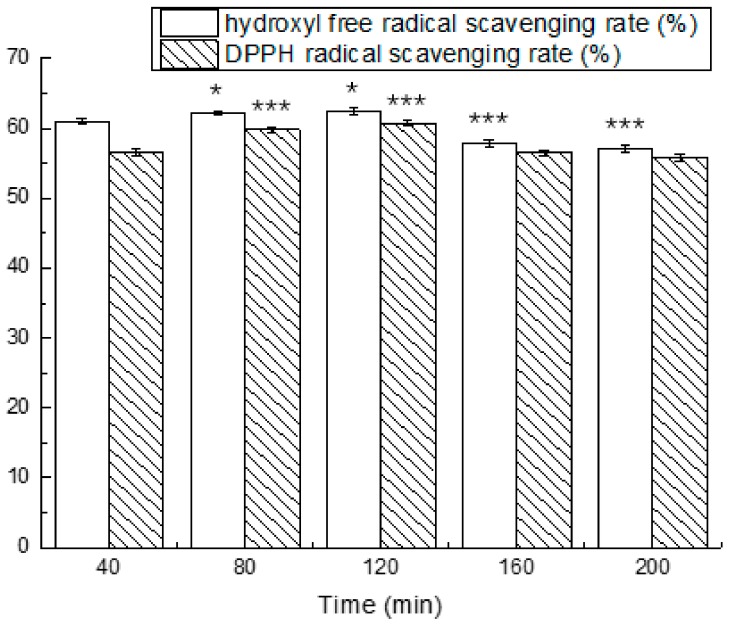
Effects of simulated intestinal fluid on the hydroxyl free radical scavenging rate and DPPH radical scavenging rate at different times. The asterisks (*) indicate significant differences (*** *p* < 0.001, extremely significant; * *p* < 0.05, significant.) of antioxidant activity in the simulated intestinal fluid during different times compared to 40 min. Mean values (*n* = 3) ± standard error are shown.

We isolated and purified the antioxidant peptides and evaluated their stability in simulated gastrointestinal fluids at different periods of time. The results are shown in Figure 5 and Figure 6.

The results from the exposure of antioxidant peptides to simulated conditions of the stomach demonstrated a changed antioxidant activity. The antioxidant activity in simulated gastric fluid for 40 min were significantly higher compared to 0 min. The scavenging rate of the hydroxyl free radical and DPPH radical at different times ranged from 92.59 ± 0.45% to 96.44 ± 0.55% and 74.86 ± 0.66% to 78.13 ± 0.38%, respectively. After exposure to simulated intestinal fluid for 40 min, the hydroxyl free radical scavenging rate decreased to 60.99 ± 0.46%, which was reduced by 34.02%, and the DPPH radical scavenging rate was reduced by 13.49%. The antioxidant activity eventually reached the maximum in 120 min.

## 4. Discussion

A great amount of antioxidant peptides is released from goat milk by fermentation. Lactic acid bacteria and yeasts are employed to produce antioxidant peptides during fermentation [19]. The strain *L. casei* L61, with high antioxidant peptides production capacity, was screened and selected as a starter culture to increase the antioxidant activity of fermented goat milk. It was reported that *L. casei* was a high antioxidative bacterial strain [20,21].

Conditions promoting the production of antioxidant peptides were optimized once the starter and the substrate were determined. A study by Shu et al. [22] showed that the optimal process parameters for producing antioxidative peptides from goat casein hydrolysate were pH of 8.9 at 62.5 °C with a hydrolysis time of 173 min. The optimum conditions for the production of antioxidant peptides from goat placenta powder with pepsin were investigated [23]. The antioxidant activity was significantly affected by the hydrolysis temperature, pH, and enzyme-to-substrate ratio.

The culture conditions for lactic acid bacteria growth are strict and generally require a carbon source, a nitrogen source, inorganic salts, and prebiotics [24]. These nutrients are not only conducive to the growth of bacteria, but also promote the production of proteases by lactic acid bacteria. Therefore, it is necessary to optimize the composition of a fermentation mixture. However, there are few studies focused on raising the yield of antioxidant peptides by adding nutrients during fermentation. Our research suggests that casein peptone, glucose, and calcium lactate had significant effects on the antioxidant peptides production from goat milk fermented by *L. casei* L61.

Calcium lactate has a promoting effect on the antioxidant activity. It was reported that ionic calcium could increase the yield of ACE inhibitory peptides when using *L. casei* as a starter during fermentation [25]. The promoting effect of calcium lactate on bioactive peptides is strong and can be attributed to the promotion of *Lactobacillus* growth. Addition of calcium lactate could promote the growth of *Lactobacillus bulgaricus* [26]. A large amount of proteases was produced and released during *L. casei* L61 proliferation, which resulted in a high antioxidant activity.

As a monosaccharide, glucose can be absorbed directly by *L. casei* L61 and then take part in the glycolytic pathway. Thus, there is a positive impact on cell metabolism by an appropriate concentration of glucose, which induces protein hydrolyzation to produce more antioxidant peptides. However, glucose at high concentrations may result in the elevation of the cell osmotic pressure, which is destructive to *L. casei* L61 growth. Moreover, glycated casein with glucose fermented by *L. casei* 4B15 exhibited significantly higher radical scavenging activities than intact casein. This is probably attributed to the formation of chemical substances with reducing capacity, since heterocyclic compounds and hydroxyl groups were formed during the stages of the glycation reaction [27,28].

Antioxidant peptides are released from proteins fermented by *L. casei* L61, and their antioxidant activity depends on the type of the original protein [29]. Casein is the main source of active peptides in fermented milk [30]. Studies have shown that the addition of caseinate can significantly increase the release of active peptides during fermentation [31]. A casein peptone supplement increased the amount of fermentation substrates in goat milk, which leads to a higher yield of antioxidant peptides.

Since the antioxidant peptides produced in goat milk fermented by *L. casei* L61 are expected to remove free radicals in the human body, the stability of the antioxidant peptides in a simulated gastrointestinal tract was evaluated. These experiments revealed a slow increase of the hydroxyl free radical scavenging rate in simulated gastric conditions during 80 min, which could be attributed to the fact that pepsin breaks down the antioxidant peptides in fermented goat milk into smaller peptides with antioxidant activity. The antioxidant activity was decreased after exposure to simulated intestinal fluids, indicating that more small peptides without antioxidant activity were generated. A study by You et al. [32] suggested that more peptide bonds were broken using pancreatin digestion than pepsin digestion.

## 5. Conclusions

In this study, the effects of the addition of a nutrients formula on the antioxidant activity of goat milk fermented with *L. casei* L61 were investigated by P–B design and RSM. In addition, experiments of simulated gastrointestinal digestion were performed to evaluate the stability of the antioxidant peptides from fermented goat milk. The results showed that calcium lactate, glucose, and casein peptone had significant effects on the antioxidant activity of fermented goat milk, and the optimal composition of the nutrient formula was found to be calcium lactate 0.99% (*w*/*v*), glucose 0.21% (*w*/*v*), and casein peptone 0.29% (*w*/*v*). The hydroxyl radical scavenging rate increased significantly from 56.50 ± 0.57% to 88.01 ± 0.69%, and the DPPH radical scavenging rate reached 63.48 ± 1.22% from 41.97 ± 0.72% under the optimal conditions. Both responses were close to the predicted values, which indicated the effectiveness of the model. In addition, the antioxidant activity remained high after incubation in simulated gastrointestinal tract conditions. The optimal nutrient formula supplement could be a good reference for preparing antioxidant peptides from goat milk.

## Figures and Tables

**Table 1:** Factors and coded levels of the Plackett–Burman (P–B) design for peptide production-promoting nutrients.

Factors	X1	X5	X6	X7	X8	X9	X10
Variables	Casein	Casein Peptone	Glucose	Soybean Peptone	Inulin	Calcium Lactate	Cysteine
	(%)	(%)	(%)	(%)	(%)	(%)	(%)
1	0.25	0.875	0.75	0.625	0.5	0.625	1.25
−1	0.2	0.7	0.6	0.5	0.4	0.5	1

X1, X5, X6, X7, X8, X9, X10 represent casein, casein peptone, glucose, soybean peptone, inulin, calcium lactate, and cysteine, respectively, while X2, X3, and X4 are three error terms.

**Table 2:** Factors level-coding table of the Box–Behnken (B–B) design for peptide production-promoting nutrients.

Factors	A: Calcium Lactate (%)	B: Glucose (%)	C: Casein Peptone (%)
−1	0.9	0.1	0.2
0	1.0	0.2	0.3
1	1.1	0.3	0.4

**Table 3:** P–B design and results for peptide production-promoting nutrients.

Factors	X1	X2	X3	X4	X5	X6	X7	X8	X9	X10	Y_1_	Y_2_
1	1	−1	1	−1	−1	−1	1	1	1	−1	77.66	55.82
2	1	1	−1	1	−1	−1	−1	1	1	1	83.34	62.79
3	−1	1	1	−1	1	−1	−1	−1	1	1	76.26	50.63
4	1	−1	1	−1	−1	1	−1	−1	−1	1	71.91	50.02
5	1	1	−1	1	1	−1	1	−1	−1	−1	71.91	50.02
6	1	1	1	1	1	1	−1	1	−1	−1	76.36	50.82
7	−1	1	1	−1	−1	1	1	−1	1	−1	76.66	55.12
8	−1	−1	1	1	1	−1	1	1	−1	1	75.61	48.96
9	−1	−1	−1	1	1	1	−1	1	1	−1	76.11	50.17
10	1	−1	−1	−1	1	1	1	−1	1	1	75.86	49.37
11	−1	1	−1	−1	−1	1	1	1	−1	1	75.02	48.29
12	−1	−1	−1	−1	−1	−1	−1	−1	−1	−1	76.51	51.77

**Table 4:** Design and results of the steepest ascent experiment for nutrients promoting peptide production.

Step	Calcium Lactate (%)	Glucose (%)	Casein Peptone (%)	Y_1_ (%)	Y_2_ (%)
1	0.6	0.6	0.7	79.65 ± 0.57%	47.43 ± 0.39%
2	0.7	0.5	0.6	80.55 ± 0.61%	49.72 ± 0.48%
3	0.8	0.4	0.5	79.69 ± 0.51%	50.14 ± 0.36%
4	0.9	0.3	0.4	80.78 ± 0.44%	61.24 ± 0.35%
5	1.0	0.2	0.3	85.43 ± 0.32%	64.56 ± 0.62%
6	1.1	0.1	0.2	81.48 ± 0.49%	60.31 ± 0.37%

Mean values (*n* = 3) ± standard error are shown.

**Table 5:** B–B experimental design and results for nutrients promoting peptide production. A, calcium lactate; B, glucose; C, casein peptone.

Runs	A	B	C	Y_1_ (%)			Y_2_ (%)		
				Actual Value	Predicted Value	Residual	Actual Value	Predicted Value	Residual
1	0	1	−1	86.84	86.67	0.07	52.92	51.68	1.24
2	1	0	−1	85.42	85.00	−0.42	59.59	59.74	−0.15
3	0	0	0	87.86	88.14	−0.28	64.51	63.89	−0.62
4	1	1	0	83.56	84.15	−0.59	51.9	52.99	−1.09
5	1	0	1	78.23	78.52	−0.29	50.08	49.91	0.17
6	−1	−1	0	85.72	85.14	0.58	55.72	54.63	1.09
7	−1	1	0	85.38	85.83	−0.45	58.3	59.37	−1.07
8	1	−1	0	82.12	81.67	0.45	64.92	63.86	1.06
9	0	0	0	87.86	88.14	0.66	63.67	63.89	−0.22
10	0	0	0	88.69	88.14	0.55	63.5	63.89	−0.39
11	−1	0	1	84.92	85.34	−0.42	54.71	54.57	0.14
12	0	1	1	85.73	84.86	0.87	47.12	46.20	0.95
13	0	−1	−1	84.64	85.51	−0.87	52.08	53.00	−0.92
14	0	−1	1	82.69	82.86	−0.17	49.76	51.00	−1.24
15	−1	0	−1	83.61	83.33	0.28	52.04	52.22	−0.18

A, B, and C represent calcium lactate, glucose, and casein peptone.

**Table 6:** ANOVA of the response variables for the hydroxyl free radical scavenging rate (Y_1_) and the DPPH radical scavenging rate (Y_2_).

Source	Y_1_				Y_2_		
	DF	MS	F	Pr > F	MS	F	Pr > F
Model	9	10.50	14.45	0.0045 **	54.11	26.73	0.0010 **
A	1	13.26	18.26	0.0079 **	4.09	2.02	0.2145
B	1	5.02	6.92	0.0465 *	18.73	9.25	0.0287 *
C	1	9.99	13.75	0.0139 *	27.98	13.82	0.0138 *
AB	1	0.79	1.09	0.3442	60.84	30.05	0.0028 **
AC	1	18.06	24.86	0.0042 **	37.09	18.32	0.0079 **
BC	1	0.18	0.24	0.6430	3.03	1.50	0.2759
A^2^	1	31.82	43.81	0.0012 **	5.99	2.96	0.1459
B^2^	1	3.74	5.14	0.0727	88.98	43.95	0.0012 **
C^2^	1	17.16	23.62	0.0046 **	267.66	132.21	<0.0001 ***
Residual	5	0.73			2.02		
Lack of fit	3	1.06	4.61	0.1835	3.18	10.87	0.0854
Pure error	2	0.23			0.29		
Cor Total	14						

*** *p* < 0.001, extremely significant; ** *p* < 0.01, very significant; * *p* < 0.05, significant. DF refers to degrees of freedom, MS refers to mean square, F and Pr > F refer to F and *p*-values, respectively.
